# Definitive Radiotherapy as a Treatment for Presumed Brainstem Meningioma Causing Collet–Sicard Syndrome in Dogs: A Case Series

**DOI:** 10.1111/vru.70110

**Published:** 2025-11-22

**Authors:** Silvia Caeiro, Juan Carlos Serra, Megan Madden, Magdalena Parys

**Affiliations:** ^1^ Hospital For Small Animals, Royal (Dick) School of Veterinary Studies University of Edinburgh Midlothian UK

**Keywords:** condylojugular syndrome, hypoglossal canal, jugular foramen, radiotherapy, VMAT

## Abstract

Collet–Sicard syndrome (CSS) is a rare neurological condition characterized by concomitant dysfunction of cranial nerves (CNs) IX–XII, resulting in variable combinations of dysphagia, dysphonia, and tongue paresis or paralysis. This retrospective case series describes three dogs diagnosed with CSS secondary to a suspected brainstem meningioma that were treated with definitive radiotherapy (RT). All dogs received volumetric‐modulated arc therapy with a total dose of 50 Gy (20 × 2.5 Gy fractions). Supportive medical management was maintained during and after RT. Clinical signs varied among dogs depending on the severity of CN involvement, and all showed clinical improvement after RT. Two dogs who achieved stable disease post‐RT were euthanized due to progressive clinical signs at 344 and 421 days post‐RT, while one dog who achieved partial response post‐RT remains alive with sustained clinical improvement at 652 days. No early or late adverse effects were recorded. This case series describes the use of RT, follow‐up advanced imaging, and medical management for the treatment of CSS in three dogs with suspected neoplastic causes.

**Abbreviations**
CN: cranial nerve
CSS: Collet–Sicard syndrome
CT: computed tomography
CTV: clinical target volume
GTV: gross tumor volume
MRI: magnetic resonance imaging
PTV: planning target volume
RT: radiotherapy

## 1 Introduction

Collet–Sicard syndrome (CSS), also termed condylojugular syndrome, is a rare cranial neuropathy caused by lesions affecting the jugular foramen and hypoglossal canal, resulting in the combined dysfunction of cranial nerves (CNs) IX (glossopharyngeal nerve), X (vagus nerve), XI (accessory nerve), and XII (hypoglossal nerve) [1, 2, 3, 4, 5, 6, 7, 8]. CSS is rarely described in humans, and based on a literature search [database search in March 2025 using PubMed terms “Collet‐Sicard syndrome AND (dog OR canine), Collet Sicard AND (dog OR canine)”], it has not yet been described in dogs. Recently, detailed neurological and imaging findings, including those of dogs from this study, were published by one of the authors [1, 2, 9, 10, 11, 12, 13, 14, 15, 16, 17].

CSS is a clinical variant of Vernet's syndrome, a cranial neuropathy caused by lesions involving the jugular foramen, and thus CNs IX–XI, for which there are sporadic reports in dogs [6, 7, 8]. The spread of pathology from the jugular foramen to the neighboring hypoglossal canal, and thus CN XII, results in its reclassification as CSS. Vernet's syndrome, also known as jugular foramen syndrome, has been described in a dog secondary to an osteoma of the tympanic bulla [6]. Despite medical management, survival time was limited to 3 weeks postdiagnosis [6]. Another study reported the computed tomography (CT) findings in five dogs with single intracranial, extra‐axial masses involving the jugular foramen that were treated conservatively [7]. Survival times in three of the dogs were less than 3 months, while the remaining two dogs were alive at a 3‐month follow‐up. Notably, one of the authors has recently described 14 dogs with clinical variations of Vernet's syndrome, including the three dogs in this case series, which had intracranial, extra‐axial masses extending from the jugular foramen to the level of the hypoglossal canal and clinical signs consistent with CSS [8]. Overall, veterinary studies detailing the treatment and prognosis for intracranial masses causing CSS are lacking.

In humans with CSS, etiologies include vascular causes, skull base trauma, metastatic tumors, iatrogenic complications, and infectious or inflammatory conditions [2, 3, 4, 5, 6, 7, 8, 9]. Primary intracranial tumors, including glomus tumors, schwannomas, and hemangiopericytomas, among other tumors, are rare causes [2, 9, 10, 16]. Clinical presentation varies based on the affected nerves. Glossopharyngeal nerve dysfunction can result in dysphagia, loss of tongue sensation, reduced salivary secretion, and impaired gag reflex. Vagus nerve involvement leads to soft palate paralysis, uvular deviation, and dysphonia. Accessory nerve damage causes atrophy of the sternocephalicus, trapezius, cleidocephalicus, and omotransversarius muscles. Hypoglossal nerve dysfunction results in unilateral tongue weakness/paresis and atrophy [1, 2, 9, 10, 11, 12, 13, 14, 15, 16, 17, 18]. Treatment depends on the underlying etiology [1, 2, 9, 10, 11, 12, 13, 14, 15, 16, 17, 18]. For patients with primary tumors, surgical excision is the treatment of choice, but its feasibility is often limited by anatomical constraints [2, 9, 10]. A case report described a child with CSS secondary to meningioma, who underwent three consecutive surgeries [2]. Due to the challenging location, palliation with medical management and temporary enteral nutrition remains the primary focus [2, 9, 10, 11, 12, 13, 14, 15, 16, 17, 18]. Corticosteroids are commonly used to help reduce tumor‐associated edema [9, 10]. A case report demonstrated clinical improvement in CSS secondary to paraganglioma following surgery with further improvement after acupuncture, electrical stimulation, vitamin B1 and B12 supplementation, and daily rehabilitation therapy [16]. Isolated case reports have reported the efficacy of stereotactic radiosurgery and fractionated radiotherapy (RT) in treating primary tumors affecting the jugular foramen and hypoglossal canal [3, 4, 5, 9, 11]. RT is primarily used for the treatment of metastatic lesions. Prognostically, vascular and infection‐related CSS cases tend to have more favorable outcomes, followed by trauma‐related cases, whereas tumor‐associated CSS generally has a poorer prognosis [2, 9, 10, 11, 12, 13, 14, 15, 16, 17, 18].

Meningiomas are the most common intracranial tumors in dogs. Diagnosis is often presumptive based on magnetic resonance imaging (MRI), due to its high accuracy, in addition to the reduced costs and risks compared to surgical biopsy [19, 20]. RT has been described as the treatment of choice, being more effective than surgical excision or palliative care alone [21, 22]. With RT, median survival times range from 1 to 2.5 years [21, 22, 23, 24, 25, 26, 27, 28, 29]. RT outcomes in dogs with CSS due to meningioma are unknown. The combination of CNs involved has the potential to lead to clinical signs that can drastically impact a patient's welfare due to difficulties in eating and swallowing and to the risk of aspiration pneumonia.

The purpose of this study was to describe the treatment of three dogs with CSS secondary to suspected meningioma with definitive‐intent RT. By evaluating clinical outcomes and survival, this study aims to provide insights into the role of RT as a viable treatment option for this rare condition in dogs.

## 2 Materials and Methods

This retrospective case series included dogs diagnosed with CSS secondary to suspected meningioma based on imaging findings that underwent definitive‐intent RT between 2022 and 2024 at the University of Edinburgh [7, 8, 19]. A neurological examination performed by a board‐certified neurologist or neurology resident in training was consistent with a polycranial neuropathy with variable involvement of CNs IX, X, XI, and XII in each dog. A radiological diagnosis of a brainstem meningioma involving the jugular foramen and hypoglossal canal was made following MRI in all dogs. Medical records were reviewed to collect data on patient signalment, medical history, clinical presentation, staging results, prior, concurrent, and subsequent therapies, response to RT using Response Evaluation Criteria in Solid Tumors (RECIST), clinical response, adverse events, and follow‐up.

MRI was performed as part of the initial diagnostic workup and reviewed by a board‐certified radiologist. Following diagnosis, a pre‐ and postcontrast CT of the head was acquired for RT planning; imaging specifications are summarized in Supporting Information S1. Patients were anesthetized and immobilized using a thermoplastic mask (Oncology Imaging Systems Ltd) over a bite plate system, Aquaplast thermoplastic mold (Adapt‐It, Qfix, Advena Ltd, PA, USA), all attached to the Varian treatment couch with an indexable U‐frame. All dogs were positioned in sternal recumbency.

RT plans were made on precontrast CT images using treatment planning software (Varian Eclipse 16.2, Varian Medical Systems, Palo Alto, CA). CT and MRI images were co‐registered, incorporating pre‐ and postcontrast CT, CT bone window, T1‐weighted post‐gadolinium, T2‐weighted, and fluid‐attenuated inversion recovery (FLAIR) MRI sequences in transverse, dorsal, and sagittal planes. Gross tumor volume (GTV) was delineated using CT and MRI data by a board‐certified radiation oncologist. Clinical target volume (CTV) ranged from 0 to 3 mm, determined at the clinician's discretion. A 2‐mm isotropic three‐dimensional expansion was applied to CTV to define the planning target volume (PTV). Organs at risk included the intracalvarial brain volume minus GTV, tympanic bullae, and eyes. A structure labelled “A Brain” was created, defined as Brain − (PTV + 2 mm), to optimize dose reduction to healthy brain tissue.

Inverse planning was used to generate volumetric‐modulated arc therapy (VMAT) with a single full arc. Heterogeneity corrections were applied, and all plans had a single isocenter. Treatment planning aimed to deliver 95% of the prescribed dose to at least 95% of the PTV. Dose calculations were performed using the AAA algorithm, and quality assurance (QA) was conducted via diode array (MapCheck3, Sun Nuclear Corporation, Melbourne, FL). The QA passing threshold was set at a minimum 98% gamma passing rate (3 mm distance‐to‐agreement, 3% absolute dose difference). RT was delivered using a 6‐MV photon beam from a Vital Beam Linear Accelerator (Varian Medical Systems, Inc., Palo Alto, CA). Kilovoltage cone‐beam CT imaging was acquired daily for patient positioning verification. The RT protocol included 20 daily fractions (Monday–Friday) of 2.5 Gy, totaling 50 Gy to the PTV.

Anesthetic protocols were tailored to each dog by a board‐certified anesthesiologist. Supportive medical therapy before, during, and after RT was adjusted according to each dog's clinical presentation. Follow‐up imaging (CT/MRI) was recommended every 3 months post‐RT for 18 months, then every 6 months. Owners were contacted at 1, 3, and 6 weeks post‐RT. Adverse events were retrospectively classified using Veterinary Radiation Therapy Oncology Group version 2 criteria [30], and disease progression was assessed clinically and neurologically. Tumor response was evaluated with RECIST when available [31].

## 3 Results

### 3.1 Patient Signalment and Diagnosis

Three female neutered dogs were included in the case series, aged 10.9, 9.6, and 10.7 years and weighing 11.7, 18.9, and 15.7 kg, respectively. The breeds represented were West Highland White Terrier, Springer Spaniel, and Staffordshire Bull Terrier, respectively (Table 1).

**TABLE 1:** Summary of patient's signalment, pre‐RT neurological examination, MRI, and CT scan findings before RT treatment.

Patient	Dog 1	Dog 2	Dog 3
Signalment	10.9 years old, FN West Highland White Terrier 11.7 kg	9.6 years old, FN Springer Spaniel 18.9 kg	10.7 years old, FN Staffordshire Bull Terrier 15.7 kg
Chief complaint	Lethargy, gagging, hypersalivation, vacant/collapse episodes	Chronic cough with acute onset of gagging and retching, increased upper respiratory sounds (stridor)	Hoarse voice, head tilt, chronic cough, facial twitching, dysphagia, retching
Duration of clinical signs before referral to RT	12 and 1.5 months of staring episodes	7 months	7 months of hoarse voice and 3 months of remaining clinical signs
Pre‐RT neurological examination	Equivocal R‐sided; head tilt; mild vestibular ataxia, R‐sided tongue atrophy; R‐sided xeromycteria; saliva accumulation	Unilateral cervical muscle atrophy; query L‐sided tongue paresis	Mild L‐sided head tilt; vestibular ataxia; postural reaction deficits in L PL; reduced gag reflex; L‐sided tongue paresis and atrophy, L‐sided myokymia
Neurolocalization based on NE and MH	Cranial nerves VII, VIII, IX, X, and XII, OR the brainstem.	Cranial nerves IX, X, XI, and XII, OR the brainstem.	Cranial nerves VII, VIII, IX, X, and XII, OR the brainstem.

Abbreviations: FN, female neutered; MH, medical history; NE, neurological examination; PL, pelvic limb.

### 3.2 Clinical Signs and Neurological Examination

All dogs had clinical signs related to a deficit of CNs IX–XII. The most common clinical signs observed were gagging (*n* = 2), retching (*n* = 2), and chronic coughing (*n* = 2). Additionally, single chief complaints included dysphagia, hypersalivation, increased upper respiratory noises, voice changes/hoarseness, facial twitching, lethargy, and vacant/collapse episodes. Neurological examination revealed tongue atrophy and/or paresis in all dogs. Other neurological deficits included vestibular ataxia (*n* = 2), head tilt (*n* = 2), cervical muscle atrophy (*n* = 1), reduced gag reflex (*n* = 1), xeromycteria (*n* = 1), and postural reaction deficits in the pelvic limbs (due to a previous diagnosis of intervertebral disc disease) (*n* = 1) (Table 1). Further details on the neurological examination findings can be found in Madden et al. [8].

### 3.3 Advanced Imaging of Primary Mass

**FIGURE 1:**
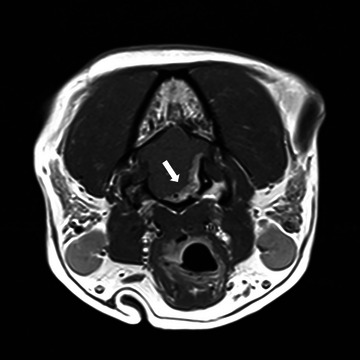
Magnetic resonance imaging of the head in Dog 1. Transverse T1‐weighted postcontrast subtraction image. Extra‐axial lesion centered around the cerebellopontine angle with extension of the lesion through the hypoglossal canal (arrow).

MRI and CT of the head were performed in all dogs. MRI findings revealed an extra‐axial lesion at the level of the cerebellopontine angle extending through the jugular foramen and hypoglossal canal (Figure 1). The lesions were well defined, irregularly marginated, and broad based, with a fusiform or plaque‐like morphology. The lesions appeared heterogeneously hyperintense on T2‐weighted and FLAIR sequences, hypo‐ to isointense on T1‐weighted images, and strongly and homogeneously contrast enhancing, consistent with a suspected meningioma, with one lesion showing a cystic component.

CT confirmed the presence of an extra‐axial mass at the cerebellopontine angle. Unilateral tongue atrophy was detected in two dogs. Mild atrophy of the ipsilateral cleidocephalicus, trapezius, sternocephalicus, and omotransverse muscles was observed in all dogs, with laryngeal muscle atrophy in two and caudal digastricus muscle atrophy in one dog. Further information on the CT and MRI findings is described in Madden et al. [8].

### 3.4 Ancillary Testing

All dogs underwent staging. Further information on investigations is described in Madden et al. [8].

### 3.5 RT

All dogs underwent 20 consecutive daily treatments from Monday to Friday for 4 weeks (with breaks during the weekend days). The median GTV was 5.0 cm^3^ (range: 2.9–6.4 cm^3^), and the median GTV as a percentage of total brain volume was 7.5% (range: 3.1%–7.9%). All RT plans met the dosimetric goal of delivering 95% of the prescribed dose to at least 95% of the PTV (Table 2).

**TABLE 2:** Volume in centimeters cubed (cm^3^) of targets and organs at risk for all dogs and radiation dose statistics for target volumes and organs at risk in centigray (cGy) for all dogs.

Dog	Volumes and dose statistic tab	Volume of targets [cm^3^] and dose to targets [cGy]			Volume of organs at risk [cm^3^] and dose to organs at risk [cGy]				
		GTV	CTV	PTV	Brain minus GTV^a^	Bulla left	Bulla right	OD	OS
1	Volume	5.1	5.1	10.8	61.4	0.5	0.5	4.8	4.8
	Min_dose_	4857	4857	4269	36	1634	1898	25	23
	Max_dose_	5207	5207	5212	5179	4699	5049	56	48
	Mean_dose_	5011	5011	5000	1895	2959	3471	36	33
	Median_dose_	5008	5008	5002	1653	2868	3475	35	32
2	Volume	2.9	2.9	7.7	90.7	1.8	1.9	6.2	6.6
	Min_dose_	4877	4877	4500	22	1604	1157	14	13
	Max_dose_	5218	5218	5228	5178	4260	3015	30	28
	Mean_dose_	5018	5018	5000	1575	2733	1771	20	19
	Median_dose_	5016	5016	5005	1347	2663	1725	20	18
3	Volume	6.4	13.2	23.7	74.4	0.9	0.9	6.3	6.3
	Min_dose_	4846	4846	4751	52	2255	1945	27	28
	Max_dose_	5145	5249	53,345	5348	4882	4121	58	65
	Mean_dose_	4956	4973	4999	2464	3383	2808	38	43
	Median_dose_	4957	4973	4995	2229	3306	2773	34	43

Abbreviations: GTV – gross tumor volume, CTV – clinical target volume, PTV – planning target volume, OD – right eye, OS – left eye.

^a^Included cerebrum, cerebellum, and brain stem.

### 3.6 Supportive Medical Management

All dogs received prednisone during (0.5 mg/kg PO SID) and after RT (0.35–1.0 mg/kg PO SID) to manage clinical signs. Gabapentin was also administered to all dogs throughout the treatment period (5.0–15.0 mg/kg PO BID to TID). In one dog, gabapentin was replaced with pregabalin (3.0 mg/kg PO BID) 25 days post‐RT due to persistent gagging, as pregabalin may benefit glossopharyngeal neuralgia [32]. Intermittent treatments like paracetamol (10.0 mg/kg PO BID–TID), maropitant (2.0 mg/kg PO SID), cisapride (0.1 mg/kg PO SID), omeprazole (1 mg/kg PO BID), ondansetron (0.5 mg/kg PO BID), and sucralfate (500 mg/dog PO SID) were given before, during, and after RT to improve gagging, hypersalivation, and retching.

### 3.7 Adverse Events, Follow‐Up, and Outcome

All dogs completed the full RT protocol. No acute or late toxicities were reported [30].

**FIGURE 2:**
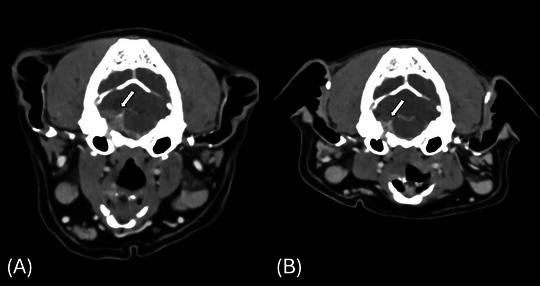
Transverse postcontrast computed tomographic images of the head in Dog 1. These images illustrate an intracranial mass, suspected meningioma, invading the jugular foramen and hypoglossal canal. Assessment of response to radiotherapy prior to treatment (A, arrow) and 97 days following radiotherapy (B, arrow).

- Dog 1: At 97 days post‐RT, follow‐up CT revealed a 30% volume reduction (partial response), with clinical signs mostly resolved [31] (Figure 2). At 168 days, the dog presented with acute gagging, hypersalivation, trembling, and falling after the reduction of prednisone. Repeat CT and neurological examination showed stable disease, and signs improved with medical management. A follow‐up CT at 291 days showed no progression and significant clinical improvement. The dog is still alive and clinically well with sustained clinical improvement. The last CT at 466 days post‐RT and MRI at 652 days showed stable disease.
- Dog 2: At 96 days post‐RT, follow‐up CT revealed stable disease with much improved gagging, retching, coughing, and upper respiratory signs. At 101 days, the dog had increased gagging and was managed medically. The dog was euthanized 344 days due to worsening of gagging and coughing, which occasionally led to vomiting, as well as respiratory noises, with no imaging or postmortem performed.
- Dog 3: At 99 days post‐RT, follow‐up CT revealed stable disease with improved clinical signs. At 220 days, the dog developed worsening facial twitching, gagging, and retching, and was managed medically. The dog was euthanized 421 days due to continued deterioration, with no imaging or postmortem performed.

## 4 Discussion

This retrospective case series aimed to describe the clinical benefits and outcomes of dogs with presumptive intracranial meningioma causing CSS treated with definitive‐intent RT.

CT and MRI revealed suspected meningiomas infiltrating the jugular foramen and hypoglossal canal, affecting CNs IX–XII, leading to severe neurological deficits and poor quality of life. At the time of diagnosis, euthanasia was strongly considered by owners. In dogs with Vernet's syndrome, medical management alone provides limited efficacy with survival times of weeks to months [6, 7, 8]. All dogs in our study demonstrated clinical improvement following RT.

Treatment with corticosteroids plays a crucial role in reducing the inflammation and edema associated with intracranial tumors. Prednisolone was given before, during, and after RT. In addition, supportive medical therapy was intermittently administered to manage clinical signs associated with the neuropathies. Survival times were similar to what has been previously described for dogs with intracranial meningiomas treated with RT [22, 23, 24, 25, 26, 27, 28, 29], with one dog still alive 652 days post‐RT, and the other two euthanized at 344 and 421 days due to neurological deterioration. These survival times exceed those with medical management alone in dogs with Vernet's syndrome secondary to intracranial, extra‐axial masses [6, 8].

The RT protocol and treatment technique described in this case series have been used for dogs with intracranial masses [22, 26, 27]. In comparison to other definitive protocols described in dogs, our protocol was chosen to optimize tumor control while minimizing the risk of radiation‐induced acute and late toxicity, based on the biological effective dose. All dogs demonstrated clinical improvement post‐RT, reinforcing the therapeutic efficacy of this protocol.

No acute or late RT‐induced toxicities were observed [30]. Dog 1 experienced clinical deterioration with recurrent gagging 168 days post‐RT, despite stable disease on CT. Clinical signs improved following an increased steroid dosage. The underlying cause remains unclear but was likely related to transient inflammation surrounding the brain mass and affected nerves, or a cerebrovascular event. In Dogs 2 and 3, progressive disease was suspected at the time of euthanasia; however, confirmation via imaging or postmortem examination was not performed.

MRI remains a valuable diagnostic tool for brain tumors and has been reported to have a specificity of 94.9% for canine meningioma [19]. However, histopathological confirmation is required for a definitive diagnosis [19, 20]. Consequently, it is possible that one of the dogs in our case series had a different condition. Since neither biopsies nor necropsies were performed on either dog, the possibility of a misdiagnosis cannot be ruled out. This highlights a limitation of our study, as MRI served as the primary diagnostic tool.

This study provides novel insights into the efficacy of definitive‐intent RT for canine CSS secondary to suspected meningioma, demonstrating favorable responses on follow‐up imaging and an excellent long‐term prognosis compared to medical treatment alone.

In conclusion, this case series suggests that definitive‐intent RT offers meaningful clinical improvement and potential for prolonged survival in affected dogs when the underlying cause is neoplastic. Further research is warranted to refine treatment protocols and optimize outcomes for dogs with CSS secondary to presumed meningioma.

## Author Contributions

Conception and design: Magdalena Parys, Silvia Caeiro, Juan Carlos Serra, and Megan Madden. Acquisition of data: Silvia Caeiro, Magdalena Parys, Juan Carlos Serra, and Megan Madden. Analysis and interpretation of data: Magdalena Parys, Silvia Caeiro, Juan Carlos Serra, and Megan Madden. Drafting of the article: Magdalena Parys, Silvia Caeiro, Juan Carlos Serra, and Megan Madden. Reviewing the article for intellectual content: Magdalena Parys, Silvia Caeiro, Juan Carlos Serra, and Megan Madden. Final approval of the completed article: Magdalena Parys, Silvia Caeiro, Juan Carlos Serra, and Megan Madden. Agreement: Magdalena Parys, Silvia Caeiro, Juan Carlos Serra, and Megan Madden.

## Disclosure

This study was previously presented as an oral presentation at the British Veterinary Neurological Society (BVNS) symposium in March 2022 and as a poster titled “Vernet's syndrome secondary to suspected meningioma in dogs” at ESVONC 2024. In addition, the clinical details and advanced imaging findings of all cases described in this study have been included in the manuscript “Jugular foramen syndrome: concurrent neurological deficits, advanced imaging findings, underlying diagnoses, and outcomes in 14 dogs (2016‐2024)” which was accepted by the *Journal of Veterinary Internal Medicine* on March 7, 2025.

## Ethics Statement

This study received ethical approval from the Royal (Dick) School of Veterinary Studies research ethics committee, VERC (Veterinary Ethical Review Committee). The radiation therapy protocol used in these cases follows the standard of care for veterinary patients. Animal owners provided written consent for the treatment provided.

## Conflicts of Interest

The authors declare no conflicts of interest.

## Supporting information




**Supporting File 1**: vru70110‐sup‐0001‐SuppMat.docx
