# Supplementary material for: Definitive Radiotherapy as a Treatment for Presumed Brainstem Meningioma Causing Collet–Sicard Syndrome in Dogs: A Case Series
Source: Vet Radiol Ultrasound. 2025 Nov 22;66(6):e70110. doi: 10.1111/vru.70110 (PMC12640213; doi:10.1111/vru.70110)
Supplement: Supplementary file 1 — Supporting File 1: vru70110‐sup‐0001‐SuppMat.docx [file VRU-66-0-s001.docx]

**Supplementary information 1. CT and MRI acquisition parameters.**

Diagnostic MR images were acquired using a 1.5T magnetic resonance scanner (Magnetom Avanto, 1.5 Tesla MRI System, Siemens, Camberley, England). MR Calibration using a phantom (Magnetom Avanto Water Phantom, Siemens, Camberley, England) is performed daily before use. T1w post contrast images were evaluated. MRI datasets were subsequently integrated for refined tumour delineation and co-registered with pre-contrast CT images to ensure greater anatomical conformity within the treatment planning protocol. Intravenous contrast with gadoterate meglumine at 0.1 mmol/kg was used. The images were uploaded to a central picture archiving and communication system (PACS).

CT scan images for RT planning were acquired using a 64‐row multidetector computed tomography scanner (Somatom® Definition AS Siemens, Erlangen, Germany). CT Calibration using a phantom (Siemens CT Water Phantom, Erlangen, Germany) is performed once weekly. Scan settings included a pitch of 0.55, tube potential of 120 kVp, reference tube current of 96 mA, slice thickness of 1.0 mm and matrix 512 × 512. Soft tissue algorithm reconstructions were assessed for the purposes of this study (Siemens proprietary kernel J40), and window width and window level were individually adjusted by the observers to optimize identification of structures (with a default soft tissue window width 350 HU, window level 40 HU). The images were uploaded to a central picture archiving and communication system (PACS).
